# Microstructured Elastomer‐PEG Hydrogels via Kinetic Capture of Aqueous Liquid–Liquid Phase Separation

**DOI:** 10.1002/advs.201701010

**Published:** 2018-03-12

**Authors:** Hang Kuen Lau, Alexandra Paul, Ishnoor Sidhu, Linqing Li, Chandran R. Sabanayagam, Sapun H. Parekh, Kristi L. Kiick

**Affiliations:** ^1^ Department of Materials Science and Engineering University of Delaware 201 DuPont Hall Newark DE 19716 USA; ^2^ Department of Biology and Biological Engineering Chalmers University of Technology Gothenburg SE‐412 96 Sweden; ^3^ Department of Molecular Spectroscopy Max Planck Institute for Polymer Research Ackermannweg 10 55128 Mainz Germany; ^4^ Department of Biological Sciences University of Delaware Newark DE 19716 USA; ^5^ Delaware Biotechnology Institute 15 Innovation Way Newark DE 19711 USA

**Keywords:** hydrogels, liquid–liquid phase separation, micromechanical, polypeptides, resilin

## Abstract

Heterogeneous hydrogels with desired matrix complexity are studied for a variety of biomimetic materials. Despite the range of such microstructured materials described, few methods permit independent control over microstructure and microscale mechanics by precisely controlled, single‐step processing methods. Here, a phototriggered crosslinking methodology that traps microstructures in liquid–liquid phase‐separated solutions of a highly elastomeric resilin‐like polypeptide (RLP) and poly(ethylene glycol) (PEG) is reported. RLP‐rich domains of various diameters can be trapped in a PEG continuous phase, with the kinetics of domain maturation dependent on the degree of acrylation. The chemical composition of both hydrogel phases over time is assessed via in situ hyperspectral coherent Raman microscopy, with equilibrium concentrations consistent with the compositions derived from NMR‐measured coexistence curves. Atomic force microscopy reveals that the local mechanical properties of the two phases evolve over time, even as the bulk modulus of the material remains constant, showing that the strategy permits control of mechanical properties on micrometer length scales, of relevance in generating mechanically robust materials for a range of applications. As one example, the successful encapsulation, localization, and survival of primary cells are demonstrated and suggest the potential application of phase‐separated RLP‐PEG hydrogels in regenerative medicine applications.

## 1 Introduction

The heterogeneity and biophysical properties of native extracellular matrix (ECM) are essential in regulating cell behavior and guiding tissue regeneration. Microstructured hydrogels that mimic the complexity of ECM thus have emerged as useful materials for controlling material mechanical properties, diffusion of macro‐ and biomolecules, and mammalian cell behavior.^1^, ^2^, ^3^, ^4^, ^5^ A variety of evidence suggests that heterogeneity in hydrogels can promote cell growth and organization in 3D; indeed, macroporous scaffolds and hydrogels containing degradable microparticles have been shown to promote osteogenic differentiation in vitro and bone tissue formation in vivo.^6^, ^7^, ^8^ Furthermore, the mechanical properties of hydrogel materials, including stiffness, elasticity, and viscoelastic properties, impact the differentiation of mesenchymal stem cells as well as tissue regeneration.^9^, ^10^, ^11^, ^12^, ^13^ Hydrogel geometries and surface curvature also influence cell morphology and migration, thus providing additional materials handles for regulating gene expression and cell functions,^14^, ^15^ independently of bulk mechanical properties. These studies together indicate the importance of engineering hydrogel microstructures with independently tunable microscale structure and micromechanical properties for controlling cell behavior. Accordingly, multiple production strategies have been pursued, such as photopatterning,^16^, ^17^, ^18^, ^19^, ^20^ selective degradation,^16^, ^21^, ^22^ and the incorporation of microparticles^3^, ^23^ synthesized via emulsion polymerization^24^, ^25^, ^26^, ^27^ and/or microfluidic^28^ technologies. However, these methods suffer from either low throughput, as in the case of photopatterning, or multiple processing steps, as in particle fabrication.

Liquid–liquid phase separation (LLPS), a well‐known phenomenon based on the unfavorable interactions of two liquids (e.g., solutions of polymers or biopolymers), results in phase separation of solutions even at a low concentration,^29^, ^30^, ^31^ and aqueous two‐phase solutions (ATPS) have thus been widely employed for the purification of proteins, polysaccharides, and DNA.^32^, ^33^, ^34^ In addition to these purification applications, LLPS also provides a highly versatile, and as yet underexplored, platform for fabricating microstructured materials. Phase behavior can be modified by factors such as temperature, polymer molecular weight, polymer concentration, ionic strength, pH, and addition of specific salts.^31^, ^35^, ^36^ The timescales over which LLPS occurs range from seconds to minutes near the critical point,^37^ and crosslinking of the solutions within this time frame can enable the generation of microstructured hydrogel materials.^38^

In such approaches, the use of solutions with comparable kinetics of phase separation and crosslinking can yield, from ATPS, materials with microstructures of various length scales.^39^, ^40^, ^41^ Our group recently reported such methodology for the single‐step fabrication of microstructured, elastomeric hydrogels with distinct micromechanical properties, via the aqueous LLPS of resilin‐like polypeptide (RLP) and poly(ethylene glycol) (PEG) solutions with stable crosslinking in both RLP‐rich and PEG‐rich phases.^42^ The rubber‐like elasticity of RLPs, including low stiffness (0.6–2 MPa), high extensibility (≈300%), and efficient energy storage (>90% resilience),^43^, ^44^, ^45^ provides distinct mechanical properties compatible with biomaterial applications for mechanically active tissues.^46^, ^47^, ^48^, ^49^, ^50^ Despite our demonstration of the one‐step generation of microstructured elastomers, however, independent manipulation of the microstructure and mechanical properties of the previously reported RLP‐PEG hydrogels was not possible, given the interdependence of the relative kinetics of phase separation and chemical crosslinking during hydrogel formation. The crosslink density (affecting mechanical properties) and crosslinking kinetics (affecting microstructure) thus could not be independently and easily tuned.

Given the demonstrated importance of microstructure in guiding cell behavior, we thus sought to develop alternative approaches that would facilitate the formation of RLP‐based elastomeric hydrogels with independently tunable domain diameters, domain compositions, and domain mechanics. Accordingly, we developed a photo‐crosslinkable RLP‐PEG LLPS system to enable on‐demand control of microstructure in hydrogels across a range of compositions of target mechanical properties. Although photo‐crosslinking reactions have been adapted widely in the formation of microstructured materials, they have been employed largely in the production of microporous hydrogels, mesoporous organohydrogels, microparticles, and copolymerized materials.^51^, ^52^, ^53^, ^54^ The development of methods that permit selective initiation of crosslinking of both phases in an ATPS thus offer substantial new opportunities to systematically control the encapsulation of microgels within a continuous matrix, providing needed flexibility for tuning (micro)mechanical properties as well as a vast parameter space for customization.^7^, ^8^, ^55^

We report here the facile production of photo‐crosslinkable RLPs and demonstrate the feasibility of producing a photo‐crosslinked ATPS with RLP‐acrylamide (RLP‐Ac) and PEG‐acrylate (PEG‐Ac) phases. The rapid photo‐crosslinking enabled the capture of microstructures with select domain properties—diameter, composition, and mechanics—as shown by multiple microscopic and spectroscopic methods. Confocal microscopy permitted facile characterization of the average domains sizes in a hydrogel after various periods of incubation prior to crosslinking, and broadband coherent anti‐Stokes Raman scattering microspectroscopy (BCARS) was used to analyze the compositions of the hydrogel domains and continuous phases throughout the demixing process. The mechanical properties of the various hydrogels were analyzed via oscillatory rheology and compared with the rule of mixtures, and atomic force microscopy (AFM) indentation was used to confirm the variations in the micromechanical properties of the microstructured materials. Finally, our studies also demonstrated that mesenchymal stem cells can be localized in select regions of the cell‐compatible scaffolds.

## 2 Results and Discussion

### 2.1 Design and Synthesis of Photo‐Crosslinkable RLP‐Ac

**Figure 1:**
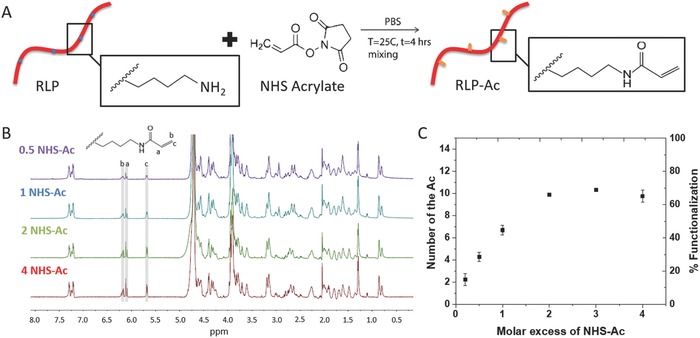
Acrylamide functionalization of RLPs. A) Schematic of RLP functionalization. Lysine residues along the polypeptide chain were reacted with an acrylic acid N‐hydroxysuccinimide ester through simple amide bond coupling reactions. B) NMR spectrum of RLP‐Ac showing the three vinylic peaks which increase in intensity with an increase in the NHS‐Ac:lysine ratio, and C) various degrees of RLP‐Ac functionalization achieved with various NHS‐Ac:lysine molar ratios from 0.2 to 4.

The RLP employed in these studies is a 23 kDa polypeptide containing 12 repeats of the putative consensus sequence (GGRPSDSYGAPGGGN) derived from *Drosophila melanogaster* and 5 repeats of lysine‐rich bundles (GGKGGKGGKGG) that can be used for crosslinking or RLP functionalization.^56^ The RLPs were expressed following procedures extensively employed in the Kiick laboratories^56^, ^57^, ^58^, ^59^ and were functionalized with acrylamide groups to facilitate the desired on‐demand photo‐crosslinking of microscale domains. Chemical modification of RLP with N‐acryloxysuccinimide (NHS‐Ac) via reaction of lysine residues yielded RLP‐Ac (**Figure** **1**A) via the protocols detailed in the Experimental Section. The degree of modification was confirmed via ^1^H NMR. The appearance of the three vinylic peaks at δ 5.65–6.30 ppm^60^ indicated the successful functionalization of the RLP‐Ac, and comparison of the area of these peaks to that of the aromatic protons from phenylalanine (δ 7.15–7.40 ppm) permitted determination of the degree of acrylation of the RLP (Figure 1B). The degree of acrylamide functionalization can be easily varied via modulation of reaction stoichiometry, where NHS‐Ac:Lys molar ratios ranging from 0.2 to 2 yield RLP with 2 to 10 acrylamide groups (RLP‐2Ac to RLP‐10Ac, Figure 1C and **Table** **1**) per chain. Further increases in the NHS‐Ac:lysine ratio (up to a ratio of 4) did not result in any additional increase in the number of acrylamide reacted per RLP. Although there are 15 lysine residues present in each RLP chain, these are distributed in short (GGKGGKGGKGG) “domains” at regular intervals in the RLP sequence; the close proximity of the lysines in these short domains possibly results in steric hindrance that prohibits complete coupling to all lysines. Moreover, the competing hydrolysis reaction in aqueous conditions^61^ is almost certainly the origin of the plateau in the degree of functionalization of the RLP. Nevertheless, the NHS‐mediated acrylation yielded a sufficiently wide range of acrylation (2Ac to 10Ac) to test the impact of acrylation on the phase separation and mechanical properties of the crosslinked RLPs.

**Table 1:** RLP‐Ac functionalization

	# Ac per RLP		% Functionalization	
RLP‐2Ac	2.2	±0.5	14.9	±3.6
RLP‐4Ac	4.0	±0.5	26.7	±3.1
RLP‐6Ac	6.7	±0.4	44.7	±2.9
RLP‐10Ac	9.9	±0.5	66.0	±3.0

### 2.2 Liquid–Liquid Phase Separation of RLP‐Ac/PEG‐Ac Solutions

**Figure 2:**
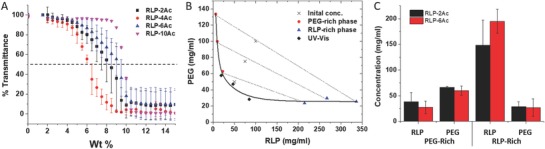
Phase separation of 50/50 RLP‐XAc/PEG‐4Ac solutions in PBS buffer. A) UV‐Vis transmittance of 50/50 RLP‐XAc/PEG‐4Ac solutions (where X was varied between 2 and 10) as a function of total polymer wt%. B) Coexistence curve for 50/50 RLP‐6Ac/PEG‐4Ac as determined by ^1^H NMR. The x indicates the initial concentration of the mixtures before phase separation. The diamond data represent the phase separation concentrations from UV–Vis data. Final concentrations after phase separation in the PEG‐rich and RLP‐rich domains are shown as circles and triangles, respectively. The dashed lines connect pairs of the PEG‐rich and RLP‐rich phases. The black line is rendered for visual clarity only. C) Comparison of concentrations in PEG‐ and RLP‐rich domains of 10 wt% 50/50 RLP‐XAc/PEG‐4Ac for X = 2 and 6.

LLPS has been observed in a variety of biopolymer solutions, such as those including gelatin and dextran. In addition, elastin‐like polypeptide solutions undergo LLPS with an increase in temperature, which is driven by a preference for homotypic self‐interactions over protein–solvent interactions,^62^ and RLP solutions of various compositions have also been demonstrated to show similar behavior. Here, the phase separation of ternary solutions of RLP‐Ac and commercially available PEG‐4Ac was confirmed via UV–vis spectrophotometric analysis (**Figure** **2**). PEG‐induced phase separation of proteins and polysaccharides is widely reported in the literature (e.g., crystallin/PEG^63^, ^64^ and BSA/PEG aqueous systems^65^) and has been a mainstay in the purification of protein‐based biopharmaceuticals.^66^ In these previous studies, as well as in our previous investigation of RLP/PEG solutions,^42^ the use of PEGs of higher molecular mass drives phase separation more strongly owing to a less favorable entropy of mixing in comparison to PEGs at similar concentrations but with lower molecular masses. However, when the PEG molecular mass is too large, the volumetric swelling is significant, thereby adding substantial, and in some cases unwanted, external stresses to the material.^67^ Therefore, 20 kDa PEG‐4Ac was selected as the second component in the RLP‐Ac LLPS system in order to balance the ability to drive phase separation while avoiding excessive swelling. Moreover, 20 kDa PEG‐4Ac gels are mechanically appropriate for soft tissue applications^67^, ^68^, ^69^ (due to the multiarm PEG molecular architecture and its moderate molecular weight). Turbidity (OD_600_) observed in 50/50 RLP‐Ac/PEG‐4Ac solutions upon phase separation was used as an indicator of LLPS and was measured at 25 °C as a function of total polymer concentration from 0 to 15 wt% (Figure 2A).

Consistent with our previous investigations in which turbidity was present for RLP‐NH_2_/PEG‐NH_2_ solutions employing 4‐arm, 20 kDa PEGs,^42^ turbidity was also observed in RLP‐Ac/PEG‐4Ac solutions for all of the various functionalized RLP‐Ac, regardless of the number of acrylamide groups per RLP chain. The observed trends in the LLPS behavior of these RLP‐Ac/PEG‐4Ac solutions were similar to those observed for the RLP‐NH_2_/PEG‐4NH_2_ solutions,^42^ i.e., with phase separation occurring at high polymer concentrations and miscibility at lower concentrations. The phase separation concentration for a given solution was defined as that at the total concentration at which the transmittance was reduced to 50%.^70^, ^71^ Differences in the number of acrylamide groups (X) conjugated per RLP chain lead to slight variation in the phase separation concentration (between 6.5 and 9.5 wt% for X between 2 and 10) as illustrated in Figure 2A, which did not show any clear trend. The phase separation concentration was also determined for solutions of RLP‐XAc (where X = 2 or 6) with PEG‐4Ac with different mixing ratios (Figure S1A–C, Supporting Information); the phase separation wt % of polymer increased from 7.7% for 25/75 RLP‐XAc/PEG‐4Ac to 11.3% for 75/25 RLP‐XAc/PEG‐4Ac.

LLPS ultimately yields two immiscible, coexisting phases at equilibrium. An understanding of the compositions of the two phases as a function of the initial concentrations of RLP and PEG permits determination of the binodal curves and tie lines, which then affords opportunities to predict phase compositions and relative phase volumes as a function of initial solution compositions. The coexistence curve for RLP‐6Ac/PEG‐4Ac solutions of different initial concentrations (10, 15, and 20 wt%) was constructed from the equilibrium concentrations of the individual separated bulk phases from 50/50 RLP‐6Ac/PEG‐4Ac solutions determined via ^1^H NMR as previously reported.^42^ The coexistence curve of RLP‐6Ac/PEG‐4Ac is shown in Figure 2B; the coexistence concentrations of RLP and PEG indicated the stronger partitioning of RLP into phase‐separated domains (with at least 9.5‐fold and up to 50‐fold greater RLP concentration than that in the PEG‐rich phase) compared to the concentration of PEG in the PEG‐rich phase (just 2.5–5 times the concentration of PEG as that in the RLP‐rich phase) (Figure 2B). We also tested if the number of acrylamides on RLP chains affects phase separation equilibria with 10 wt% RLP‐2Ac/PEG‐4Ac solutions and found statistically identical concentrations in each phase (Figure 2C). The similarity in the coexistence concentrations for the two RLP‐XAc shows that differences in the number of acrylamide groups of the RLP does not significantly affect the coexistence concentrations of RLP and PEG in the RLP‐rich and PEG‐rich phases.

The volume fraction of the of PEG‐rich (top phase I, φ^I^) and RLP‐rich (bottom phase II, φ^II^) phases can be determined from the tie line according to the lever rule^39^

(1) $$\phi^{I}=\frac{BC}{AB}$$

(2) $$\phi^{\mathrm{II}}=\frac{AC}{AB}$$

where *A* refers to the composition in the PEG‐rich phase, *B* refers to the composition in the RLP‐rich phase, and *C* is the initial composition on the phase diagram. The volume fraction of the RLP‐rich phase (φ^II^) was determined, from the coexistence curve for the 10 wt% 50/50 RLP‐XAc/PEG‐4Ac, to be 0.22 ± 0.05 for RLP‐2Ac and 0.16 ± 0.04 for RLP‐6Ac; the volume fraction of the PEG‐rich phase (φ^I^) was 0.89 ± 0.09 and 0.86 ± 0.07 for RLP‐2Ac and RLP‐6Ac, respectively. Both RLP‐2Ac and RLP‐6Ac show a lower volume fraction for the RLP‐rich phase versus the PEG‐rich phase, which will result in an RLP‐rich dispersed and PEG‐rich continuous phase. Statistical analysis from the ANOVA Tukey‐Kramer HSD test illustrates that the volume fractions of the RLP‐rich phase (*p*‐value 0.69) and PEG‐rich phase (*p*‐value 0.98) were statistically similar regardless of the number of acrylamide groups linked to the RLP.

### 2.3 Characterization of Microstructure and Bulk Mechanics of Microstructured Elastomers

**Figure 3:**
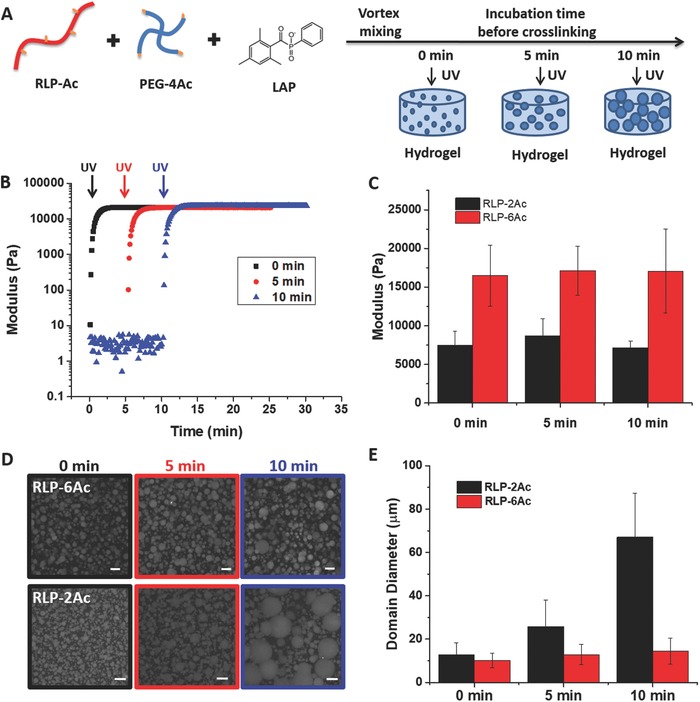
Temporally controlled microstructured hydrogels. A) Schematic of hydrogel formation and microstructure development. B) Time sweep of 10 wt% 50/50 RLP‐6Ac/PEG‐4Ac with UV irradiation at 0, 5, and 10 min after vortex mixing. C) Storage moduli comparison for RLP‐2Ac and RLP‐6Ac with UV irradiation at 0, 5, and 10 min after mixing. D) Autofluorescence images of photo‐crosslinked 10 wt% 50/50 RLP‐6Ac/PEG‐4Ac and 10 wt% 50/50 RLP‐2Ac/PEG‐4Ac hydrogels; microscale RLP‐rich domains grow in diameter when precursors were incubated at room temperature for 0, 5, and 10 min prior to photo‐crosslinking. E) Average domain diameters of the RLP‐rich domains over time for RLP‐2Ac and RLP‐6Ac solutions with PEG‐4Ac.

The possibility of employing photo‐crosslinking of the phase‐separated RLP‐Ac/PEG‐4Ac solutions to permit temporal control over the microstructure of resulting hydrogels was evaluated (**Figure** **3**A). Photochemical methods were employed to crosslink solutions of functionalized RLP‐XAc with PEG‐4Ac via incorporation of the biocompatible photoinitiator lithium phenyl‐2,4,6‐trimethylbenzoylphosphinate (LAP) and irradiation with 365 nm light;^72^, ^73^ crosslinking should therefore occur both throughout the individual phases as well as across the phase‐separated interface in the hydrogel and easily allow domains of targeted diameters to be captured during the course of phase separation.

In situ oscillatory shear rheology with a UV accessory was used to characterize photo‐crosslinking gelation kinetics upon application of UV light (365 nm, 5 mW cm^−2^, for a duration of 10 min) and to measure the shear storage moduli of the crosslinked phase‐separated RLP‐PEG hydrogels at time points 0, 5, or 10 min after vortex mixing. Time‐sweep rheology data for a 10 wt% 50/50 RLP‐6Ac/PEG‐4Ac hydrogel show in Figure 3B that the storage modulus (*G*′) increased and stabilized rapidly with gelation, which occurred in less than 30 s upon application of UV light, although irradiation for longer than 30 s resulted in corresponding increases in final storage modulus; a plateau in the storage modulus was observed within 4 min. The data illustrate that the final shear storage modulus can be modulated over the range of 1 to 17 kPa by varying the duration of UV irradiation from 30 s to 10 min (Figure S2, Supporting Information), regardless of at what point the UV irradiation is applied after mixing. In addition, there was no significant change in the gelation time or final plateau storage modulus (Figure 3C, *G*′ values of 16.5 ± 4.0, 17.1 ± 3.2, and 17.1 ± 5.4 kPa at 0, 5, and 10 min) as a function of the point at which the UV irradiation was applied, suggesting that the extent of phase separation does not alter the bulk mechanical properties of these materials although the microstructures are trapped out of equilibrium. Furthermore, the storage modulus of RLP‐2Ac/PEG‐4Ac hydrogels was similarly insensitive to the variation in times of crosslinking, with a *G*′ of ≈7.7 ± 1.7 kPa for hydrogels crosslinked at various time points after mixing (Figure 3C). The overall lower moduli in the RLP‐2Ac/PEG‐4Ac relative to the RLP‐6Ac/PEG‐4Ac hydrogels is almost certainly a result of the lower acrylamide functionality and consequently lower crosslinking density of the RLP‐2Ac compared to RLP‐6Ac.

Multiphoton microscopy was employed to image microstructures that developed in these hydrogels; visualization of the domains was enabled by the autofluorescence of the RLP phase with 755 nm excitation. Phase contrast optical microscopy confirmed that both pure RLP and pure PEG hydrogels lacked any microstructure after crosslinking (Figure S3, Supporting Information). Mixing of the 50/50 RLP‐XAc/PEG‐4Ac solutions in phosphate‐buffered saline (PBS) immediately results in an opaque solution for both RLP‐2Ac and RLP‐6Ac; the evolution of domains captured by UV crosslinking at 0, 5, and 10 min are shown in Figure 3D. In both solutions, the diameters (and distribution of diameters) of the RLP domains increased with time. The 10 wt% 50/50 RLP‐6Ac/PEG‐4Ac hydrogels developed RLP‐rich domains with an initial diameter of 10.2 ± 3.4 µm (volume fraction of 0.06) after immediate UV crosslinking; the domains increase in diameter to 14.5 ± 6.0 µm (volume fraction of 0.11) when photo‐crosslinked 10 min after mixing. By contrast, the evolution of the diameters of the RLP domains in 10 wt% 50/50 RLP‐2Ac/PEG‐4Ac solutions occurred more rapidly, with diameters of 12.8 ± 5.5 µm (volume fraction of 0.08) upon immediate crosslinking and 67.1 ± 20.3 µm (volume fraction of 0.12) when photo‐crosslinked at 10 min after mixing (Figure 3E). The domain diameter distribution data in Figure S4 of the Supporting Information also delineate the more rapid growth of RLP‐rich domains over time for the RLP‐2Ac versus RLP‐6Ac‐containing solutions.

The phase separation kinetics of both systems was distinct while the equilibrium coexistence concentrations were almost identical.^74^ The evolution of the coalescence of the domains is balanced by gravitational, flotational, and frictional forces,^75^ and thus the rate of the phase separation is a function of the relative density, interfacial tension, and viscosity of both phases, which do not influence equilibria. Given the similarities in the compositions of the phases for the RLP‐2Ac and the RLP‐6Ac, the slower rate of domain growth in the RLP‐6Ac/PEG‐4Ac solutions is thus suggested to arise from a lower interfacial tension for the RLP‐6Ac, likely as a result of increased hydrophobic interactions and miscibility between the RLP‐6Ac and the PEG‐4Ac (relative to RLP‐2Ac). Analysis of the normalized domain diameter distribution data in Figure S4 of the Supporting Information suggests a sharp asymmetric peak for the RLP‐6Ac/PEG‐4Ac samples, consistent with an Ostwald ripening model,^76^ while the RLP‐2Ac/PEG‐4Ac samples are more consistent with the Smoluchowski model, with a size distribution independent of the volume fraction^77^ (Figure S4, Supporting Information), suggesting quantitative differences in the RLP‐2Ac and RLP‐6Ac phase separation kinetics that can be exploited to generate microscale domains of various sizes in these phase‐separated hydrogels.

**Figure 4:**
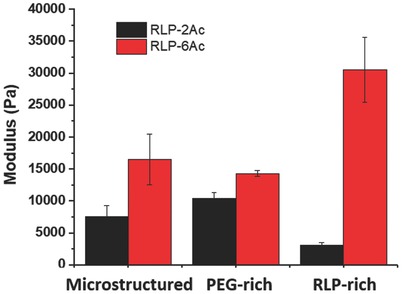
Oscillatory rheological characterization of 10 wt% 50/50 RLP‐Ac/PEG‐4Ac hydrogels. The comparison of storage moduli of microstructured hydrogels and equilibrium PEG‐rich and RLP‐rich phases for RLP‐2Ac and RLP‐6Ac.

Interestingly, as clearly indicated by the data in Figure 3C, the storage moduli of these materials were insensitive to the size of the microstructures that evolved over 0, 5, and 10 min prior to crosslinking (Figure 3D,E), which is particularly notable for the RLP‐2Ac solutions. To first order, the modulus of composite materials depends on the volume fraction and the mechanical properties of each phase according to the rule of mixtures,^78^ with the upper bound storage modulus (*G*_U_) and lower bound storage modulus (*G*_L_) related by

(3) $$G_{U}=G^{I}\;\phi^{I}+G^{\mathrm{II}}\phi^{\mathrm{II}}$$

(4) $$\frac{1}{G_{L}}=\frac{\phi^{I}}{G^{I}}+\frac{\phi^{\mathrm{II}}}{G^{\mathrm{II}}}$$

where *G* is the storage modulus and φ is the volume fraction in phase I (top, PEG‐rich) and phase II (bottom, RLP‐rich). The similarity in the moduli of these materials at different crosslinking times is consistent with the observed relatively small changes in the volume fractions of the phases over time; indeed, the fraction of the PEG‐rich phases remains at ≈0.90 over the 10 min time period of the experiments. The shear storage modulus for each phase was determined by photo‐crosslinking of each of the individual macroscale phases on the rheometer after collection of the upper or lower phases that result from overnight incubation of RLP‐XAc/PEG‐4Ac solutions. As shown in **Figure** **4**, the shear storage moduli for the crosslinked PEG‐rich phase (*G* ^I^) from the RLP‐2Ac/PEG‐4Ac and RLP‐6Ac/PEG‐4Ac solutions were 10.4 ± 0.9 and 14.3 ± 0.4 kPa, respectively; while the storage moduli for the crosslinked RLP‐rich phase (*G* ^II^) from the RLP‐2Ac/PEG‐4Ac and RLP‐6Ac/PEG‐4Ac solutions were 3.1 ± 0.3 and 30.5 ± 5.1 kPa, respectively. The higher storage modulus for the crosslinked RLP‐6Ac was expected owing to the higher crosslinking density afforded by the higher degree of acrylation for RLP‐6Ac, as noted above. By applying the rule of mixtures (Equations (3) and (4)) to these phase‐separated hydrogels, the calculated *G*′ values for RLP‐6Ac (15–17 kPa) and RLP‐2Ac (6.4–9.9 kPa) matched well with the measured values (RLP‐6Ac 16.8 ± 3.8 kPa and RLP‐2Ac 7.7 ± 1.7 kPa). The difference between the storage moduli in the RLP‐rich and PEG‐rich phases suggests that the micromechanical properties will be heterogeneous in the microstructured hydrogels, which could be used to selectively and locally promote cell proliferation and/or differentiation within the domains or matrix.^11^, ^13^, ^79^

### 2.4 Distinct Composition and Mechanics of Domains in Micostructured Elastomers

**Figure 5:**
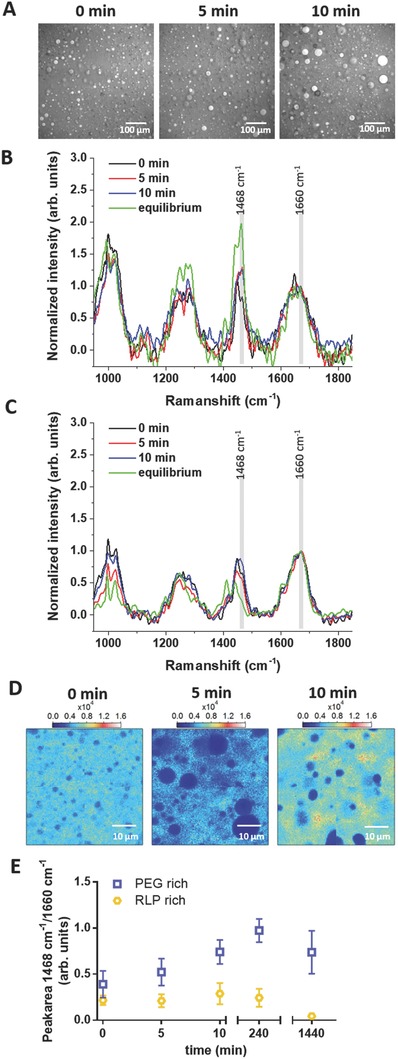
BCARS data for 10 wt% 50/50 RLP‐6Ac/PEG‐4Ac hydrogels. A) BCARS images at the asymmetric CH_3_ stretching vibration (2930 cm^−1^); highest intensity correlates with RLP‐rich domains (scale bar: 100 µm). Fingerprint RL spectra, normalized to ≈1660 cm^−1^, from B) PEG‐rich and C) RLP‐rich areas at different crosslinking times. D) Ratio images (integrated intensities at 1468/1660 cm^−1^) representing the [PEG] relative to [RLP] within the hydrogels when photo‐crosslinked at 0, 5, and 10 min. Blue to red represents a high value of [PEG]/[RLP] and blue indicates high [RLP]/[PEG] ratios (i.e., low values of [PEG]/[RLP]) (scale bar: 10 µm). E) Peak area ratios of the vibrations 1468 and 1660 cm^−1^ plotted versus crosslinking time (0, 5, and 10 min) and after 240 and 1440 min (obtained by peak fitting).

Characterization of microstructured hydrogels has been previously reported via cryogenic electron microscopy (cryo‐EM),^80^, ^81^, ^82^ small angle neutron scattering,^54^, ^80^, ^83^ and coherent anti‐Stokes Raman scattering (CARS) microscopy;^69^, ^84^, ^85^ Given the simplicity and noninvasiveness of the technique, BCARS was employed to assess the relative compositions of the domains and continuous phases of RLP/PEG solutions as a function of demixing time during the phase separation process for the 10 wt% 50/50 RLP‐6Ac/PEG‐4Ac hydrogels; representative results (images and spectra) are shown in **Figure** **5**. Raman‐like (RL) spectra were produced from the BCARS dataset collected from hydrated hydrogels, for which the intensities then are linearly related to the concentration of the polymer probed. In order to quantify the RLP and PEG content in different phases of microstructured RLP‐PEG hydrogels in situ, pure RLP, and PEG spectra (Figure S4A, Supporting Information) were first acquired to determine suitable Raman bands for selective imaging of these molecules. The RL spectra of PEG showed good correspondence with conventional Raman spectra in the fingerprint and CH‐stretching regions.^86^, ^87^ A comparison of the features of the RLP and PEG spectra indicated that a spectral ratio of $I_{1468\;{\mathrm{cm}}^{-1}}$ (δCH_2_)/$I_{1660\;{\mathrm{cm}}^{-1}}$ (Amide I) accurately captures the [PEG]/[RLP] ratio. This characteristic Raman peak ratio was used to analyze the composition of the RLP‐ and PEG‐rich regions of the RLP‐6Ac/PEG‐4Ac hydrogels as a function of incubation time. Figure 5A shows the intensity distribution of the vibration at 2930 cm^−1^, representing the density of CH_3_ bonds, which is reflective of the regions with large [RLP] due to its relatively large number of CH_3_ groups. These results show similar domain diameters as observed with multiphoton microscopy (Figure 3D); the corresponding RL fingerprint spectra of the RLP‐rich phase and PEG‐rich phase (normalized to the maximum value in the amide I region (≈1660 cm^−1^)) are shown in Figure 5B,C (full spectra, Figure S5B,C, Supporting Information) and show clear spectral differences between the RLP and PEG rich domains. Figure 5D shows a map of the RLP‐rich phase via plotting of $I_{1468\;{\mathrm{cm}}^{-1}}$/$I_{1660\;{\mathrm{cm}}^{-1}}$ in the 10 wt% 50/50 RLP‐6Ac/PEG‐4Ac hydrogels crosslinked at 0, 5, and 10 min after mixing. These BCARS images illustrate the multiphase structure of these mixtures (consistent with Figure 5A) wherein RLP‐rich domains (that still contain PEG) grow larger with time within a PEG‐rich continuous phase that becomes enriched with PEG during the phase separation process.

The 1468 cm^−1^ (δCH_2_) peak was observed to increase in the PEG‐rich phase and decrease in the RLP‐rich phase over time, indicating that the PEG polymer was partitioning out of the RLP‐rich droplets and into the PEG‐rich matrix. Plotting the peak ratio $I_{1468\;{\mathrm{cm}}^{-1}}$/$I_{1660\;{\mathrm{cm}}^{-1}}$, allowed for quantification of the phase separation with mixing time (Figure 5E), confirming previous observations (from domain size ripening) that the PEG‐rich phase appears to achieve equilibrium after 10 min. The low and relatively consistent spectral ratio observed in the RLP‐rich phase is likely a result of the high RLP concentration in the RLP‐rich phase, which may obscure changes of the $I_{1468\;{\mathrm{cm}}^{-1}}$/$I_{1660\;{\mathrm{cm}}^{-1}}$ ratio. Both the spectral data and the ratiometric analysis are consistent with the ^1^H NMR analysis (Figure 2C) and suggest that at equilibrium there is almost no PEG in the RLP‐rich domains while the PEG‐rich continuous phase contains a significant amount of RLP. The relatively constant [RLP]/[PEG] ratio in the RLP‐rich phase over the first 240 min suggests that the RLP‐rich domains first grow in size (coalesce) and then later exclude the PEG. The heterogeneous PEG‐rich phase, by contrast, appears to always contain a certain amount of RLP. In concert with photo‐crosslinking, BCARS microspectroscopy provides an important capability for facile observation of the spatial organization and chemical compositions of phases during the course of phase separation.

**Figure 6:**
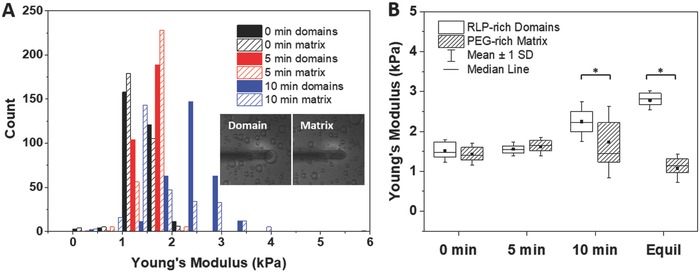
Micromechanical characterization of hydrogel domains via AFM indentation. A) The distribution of Young's moduli from indentation of RLP‐rich domains and PEG‐rich matrix for 10 wt% 50‐50 RLP‐6Ac/PEG‐4Ac hydrogels crosslinked 0, 5, and 10 min after mixing. The phase‐separated RLP‐rich domains and PEG‐rich matrix were visualized via optical microscopy and indented separately. Optical microscopy images of the RLP‐PEG thin hydrogel with the AFM probe located at the RLP‐rich domain and PEG‐rich matrix. B) The box plot shows the statistical distribution of the data for the phase‐separated domains crosslinked at 0, 5, and 10 min postmixing, as well as the mechanical properties of the individual phases photo‐crosslinked after bulk phase separation (Equil). The asterisk indicates statistically significant differences between the mean values of the marked samples and all other samples (*p* < 0.01).

The micromechanical properties of the hydrogel domains were characterized via AFM indentation. Thin hydrogels (40–60 µm) were formed to minimize optical scattering within the hydrogels to observe the domains with phase contrast microscopy and to identify target locations for indentation. An AFM tip with a 1 µm spherical probe was employed to determine the mechanical different in distinct regions within the RLP‐PEG hydrogels^88^, ^89^, ^90^ and reduce the substrate effects that would occur by probing a thin hydrogel with a large probe.^91^, ^92^ The Young's modulus determined from each indentation is presented in **Figure** **6**A, showing the distribution of values for the RLP‐rich domains and the PEG‐rich matrix for 10 wt% 50/50 RLP‐6Ac/PEG‐4Ac microstructured hydrogels crosslinked at 0, 5, and 10 min. The hydrogels photo‐crosslinked in 0 min show similar Young's moduli for both the domains and matrix, with means of 1.5 ± 0.3 kPa for the RLP‐rich domains and 1.4 ± 0.3 kPa for the PEG‐rich matrix (Figure 6B; *p* = 0.28). Likewise, the Young's moduli of the hydrogels photo‐crosslinked at 5 min show similar values. By contrast, hydrogels photo‐crosslinked at 10 min showed RLP‐rich domains (2.2 ± 0.5 kPa) that are mechanically distinct from the PEG‐rich matrix (1.7 ± 0.9 kPa; *p* < 0.01), with differences in modulus that are qualitatively consistent both with the differences observed in bulk rheology measurements of the individual phases (Figure 4) and with the difference in composition indicated by the BCARS data at later time points (Figure 5). The significantly lower Young's modulus measured from AFM indentation versus that predicted from the bulk oscillatory rheology measurement is consistent with previous studies,^93^ and likely arises from the confinement of water in a hydrogel during bulk rheological measurements; this confinement increases the resistance to deformation and consequently the modulus. By contrast, the lack of confinement, coupled with the small force applied during the AFM indentation, minimizes resistance to deformation.^93^ Although evaluation of the local water content and swelling within the domains and matrices was not possible to measure in the RLP‐PEG hydrogels, the swelling ratio for the bulk microstructured hydrogels as well as that of the crosslinked PEG‐rich phase and crosslinked RLP‐rich phase (each separately, isolated after bulk phase separation and then crosslinked) in PBS is shown in Figure S6 of the Supporting Information. The RLP‐rich phase exhibited a lower swelling ratio compared to that of the PEG‐rich phase, as might be expected based on the known swelling of PEG‐based hydrogels;^94^, ^95^ the higher water content of the PEG phase is expected to lower the mechanical properties of the PEG‐rich phase relative to that of the RLP‐rich phase. Indeed, AFM measurements of the bulk RLP‐rich and PEG‐rich bulk phases crosslinked after overnight phase separation, yield Young's moduli (2.7 ± 0.2 and 1.1 ± 0.4 kPa, respectively (indicated by the data marked Equil in Figure 6B) that are perfectly consistent with those measured from the domains and matrix in the microstructured RLP‐PEG hydrogels. These data thus illustrate the power of the combined BCARS and micromechanical measurements; the evolution of the phases and their compositions over time can be visualized and related to the micromechanical properties within the phase‐separated hydrogels.

### 2.5 Cell Viability and Growth in 3D LLPS Microstructured Elastomers

**Figure 7:**
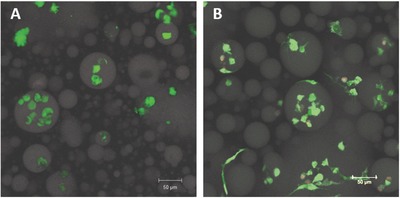
Cytocompability and cell localization in microstructured hydrogels. Confocal Z‐stack maximum intensity projections images for 3D cultures of encapsulated A,B) hMSC in 10 wt% RLP‐MMP‐RGD‐2Ac/PEG‐4Ac hydrogels at (A) day 1 and (B) day 7. Colors indicate live cells (calcein, green), dead cells (ethidium homodimer, red), and autofluorescence of RLP‐rich domains (white).

Previous reports have suggested the promise of hierarchically structured hydrogels for directing cell–matrix interactions (spreading, migration, and adhesion),^55^, ^96^, ^97^ as well as promoting mesenchymal stem cells toward osteogenic differentiation.^6^, ^7^, ^8^ Thus, the cytocompatibility of the RLP/PEG hydrogels was evaluated via 3D encapsulation of human mesenchymal stem cells (hMSC). The hydrogel precursors were first vortex mixed alone and then gently pipette mixed with the cells to minimize cell death induced by the high shear stress of vortex mixing. The rapid photo‐crosslinking of the hydrogels permitted cell encapsulation with good cell viability and cell distribution. The 10 wt% 50/50 RLP‐MMP‐RGD‐2Ac/PEG‐4Ac was used in the cell encapsulation studies owing to the wider range of domain diameters accessible; the RLP‐2Ac contained a matrix metalloproteinase (MMP)‐sensitive peptide for cell‐mediated degradation and an RGD cell‐adhesive ligand to facilitate integrin‐mediated cell attachment. Cell viability was evaluated via staining with calcein and ethidium homodimer and confocal imaging at day 1 and day 7 as illustrated in **Figure** **7**. The hMSCs maintained an exceptionally high cell viability of 95% (Figure 7A,B) within the microstructured hydrogels over a period of 7 d, with spreading clearly observed by day 7. Interestingly, the cells showed elongated morphologies *only* around the RLP‐rich domains (Figure 7B). Similar cell viability was observed for hMSCs encapsulated in RLP‐MMP‐RGD‐6Ac/PEG‐4Ac hydrogels, as shown in Figure S7 of the Supporting Information, as well as for human microvascular endothelial cells in both RLP‐6Ac and RLP‐2Ac systems (data not shown). The high viability and organization of hMSCs around the RLP‐rich domains demonstrates the promise of employing these organized RLP/PEG hydrogels to localize cells via methods that could be extended to multiple cell types and cocultures.

## 3 Conclusions

Photo‐crosslinking methods were exploited as a facile method to capture morphologically, chemically, and mechanically distinct phases in microstructured hydrogels during LLPS of RLP‐Ac/PEG‐4Ac solutions. Evaluation of the LLPS for RLPs with various degrees of acrylamide functionalization established that equilibrium phase diagrams were not significantly affected by the degree of functionalization. Photo‐triggered crosslinking of RLP‐Ac/PEG‐4Ac during the phase separation permitted the production of RLP‐PEG hydrogels with RLP‐rich domains with various diameters; a higher degree of RLP acrylation reduced the rate of domain growth, presumably by increased miscibility mediated by hydrophobic interactions between the RLP‐6Ac and PEG‐4Ac. Controllable photo‐crosslinking permits the modulation of the modulus via UV exposure time, allowing for on‐demand and independent tuning of microstructure and mechanical properties. Significant differences in the compositions (and thus mechanical properties) of the developing domains and continuous phase were indicated to occur only after 10 min of phase separation, as indicated by BCARS microscopy and AFM; interestingly, the microstructured matrices exhibit bulk mechanical properties that correspond to the rule of mixtures theory and do not vary over time. Furthermore, the materials demonstrated spatial localization of multiple cell types, at high viabilities, around RLP‐rich domains. Overall, the LLPS of RLP‐Ac/PEG‐4Ac solutions, when captured via photo‐crosslinking, permits independent tuning of the microstructure and micromechanical properties of hydrogels that can be used to design complex materials for biomedical and other applications. The high cell viability and capability to guide cell organization within the microstructured hydrogels indicates their potential use in regenerative medicine applications.

## 4 Experimental Section

*Materials*: Chemically competent cells of *Escherichia coli* strain M15[pREP4] (for transformation of recombinant plasmids) and Ni‐NTA agarose resin (for protein purification) were purchased from Qiagen (Valencia, CA). Acrylate‐terminated 4‐arm (20 kDa) PEG was purchased from Jemken. PBS was purchased from Mediatect (Manassas, VA). Deuterium oxide and NMR solvents were purchased from Cambridge Isotope Laboratories (Tewksbury, MA). All other chemicals were obtained from Sigma‐Aldrich (St. Louis, MO) or Fisher Scientific (Waltham, MA) and were used as received unless otherwise noted.

*RLP Expression and Purification*: RLP protein expression and purification was conducted as previously reported by the laboratories.^56^, ^57^, ^58^, ^59^ In brief, a single colony of *E. coli* M15[pREP4] containing the desired RLP construct was inoculated in (100 mL) sterile LB media containing antibiotics (100 µg mL^−1^ ampicillin) and grown overnight. Overnight culture media was subsequently used to inoculate (750 mL) of 2× TYmedia (yeast 10 g L^−1^, NaCl 5 g L^−1^, tryptone 16 g L^−1^) for protein expression. The (750 mL) cultures were grown in a shaker at 37 °C until the OD_600_ reached 0.6–0.8, and then isopropyl β‐D‐1‐thiogalactopyranoside (IPTG) was added a final concentration of 1 × 10^−3^ m to induce protein expression for 4 h. Cells were harvested by centrifugation (5000 rpm for 15 min at 4 °C), and the cell pellets were stored at −20 °C. The frozen cell pellets were lysed by freeze‐thaw cycles and the lysed cell pellets were suspended in pH 8.0 native lysis buffer (50 × 10^−3^ m NaH_2_PO_4_, 300 × 10^−3^ m NaCl, 10 × 10^−3^ m imidazole) with 0.45 g lysozyme. Lysed cells were further disrupted via sonication on ice, using a Fisher Scientific model 500 Sonic Dismembrator (10 mm tapered horn) for 20 min with a 10 s recovery time. The supernatant from centrifugation (10 000 rpm for 60 min) of cell lysate was collected and heated to 80 °C for 5 min to remove hydrophobic proteins by centrifugation at 500 rpm for 10 min. The supernatant was collected and adjusted pH to 8.0, followed by incubation with Ni‐NTA resin overnight at 4 °C. The resin was then loaded into a gravitational flow column, washed with native lysis buffer, native wash buffer (50 × 10^−3^ m NaH_2_PO_4_, 300 × 10^−3^ m NaCl, 20 × 10^−3^ m imidazole, pH 8.0), and finally eluted with native elution buffer (50 × 10^−3^ m NaH_2_PO_4_, 300 × 10^−3^ m NaCl, 250 × 10^−3^ m imidazole, pH 8.0). 100 mL elution fractions were carefully transferred and dialyzed (MWCO 10 kDa) against deionized (DI) water (5 L) at 4 °C with at least six changes of water before sterile filtration and lyophilization. The protein yield was ≈30–50 mg per liter of cell culture.

*Functionalization and Characterization*: The RLP proteins were functionalized with acrylamide groups via modification of regularly positioned lysine residues on the polypeptide chain. First, the RLP proteins were dissolved in PBS (10 mg mL^−1^). The acrylic acid NHS‐Ac was dissolved in dimethyl sulfoxide (DMSO) in 50 mg mL^−1^ separately and drop‐wise added into the RLP solution. The ratio of NHS‐Ac to lysine was varied depending upon the desired functionality of the conjugate. The reaction was stirred at room temperature for ≈4 h. This reaction solution was diluted eight times with DI water to prevent precipitation and dialyzed (Snakeskin, 3.5 kDa, Thermo Scientific) against DI water at 4 °C (in a cold room) to remove byproducts and DMSO. The purified RLP‐Ac was filtered and lyophilized and stored at −20 °C prior to experiment.

The functionality of the RLP‐Ac was characterized via ^1^H NMR spectrometry. The purified RLP‐Ac (≈2 mg) was dissolved in (600 μl) D_2_O (Cambridge Isotope Laboratories, Tewksbury, MA) and analyzed using an AVIII 600 MHz NMR spectrometer (Bruker Daltonics, Billerica, MA). The protons from the eight phenylalanine residues per RLP molecule were used as an internal reference for the quantification of acrylamide group functionality. The integration of the aromatic protons of phenylalanine (^1^H NMR (600 MHz, D_2_O, δ): 7.15–7.40 (m, 5H)) was compared to the integration of the vinylic protons of the acrylamide that resulted from the reaction of the acrylamide and lysine amine groups (^1^H NMR (600 MHz, D_2_O, δ): 5.65–6.30 (d, 3H)).

*Characterization of RLP‐Ac/PEG‐4Ac Phase Separation*: RLP‐Ac and PEG‐4Ac were dissolved in PBS at various concentrations from 10 to 20 wt%. The two solutions (at 50/50 RLP‐Ac/PEG‐4Ac mass ratios) were vortex mixed at room temperature. The phase diagrams were determined by heating a series of RLP/PEG solutions from 4 to 60 °C at 1 °C intervals and observing when the solutions transitioned from turbid to clear; the transition point was identified as the point of 50% transmittance. UV–vis spectroscopy was also used to characterize the concentration dependence of the phase separation at room temperature. Samples comprising 15 wt% (w/v) 50/50 RLP‐Ac/PEG‐4Ac in PBS were diluted sequentially by 0.5 wt% increments. Each solution composition was vortex mixed and the absorbance was measured at room temperature with a 10 mm path length quartz cuvette at a wavelength of 600 nm to determine the turbidity of the solution. Although phase separation was observed in all solutions comprising the PEG‐4Ac, it should be noted that variations in PEG‐4Ac viscosity were apparent in different lots of PEG‐4Ac ordered from the same manufacturer; these viscosity differences affect phase separation kinetics, so all comparisons here are made between separate samples, but those employing PEG‐4Ac of similar viscosity.

*Characterization of Equilibrium Concentrations*: The RLP‐Ac and PEG‐4Ac were dissolved into d‐PBS separately at a 10, 15, 20 wt% (w/v) (w/v) concentration. The two stock solutions were mixed at 50/50 RLP‐Ac/PEG‐4Ac mass ratios at room temperature and allowed to phase separate overnight into two immiscible layers. Samples were carefully taken from the top and the bottom layer to prevent mixing of the two liquids and were dissolved in deuterium oxide (D_2_O) that contained 0.01 mg mL^−1^ 4,4‐dimethyl‐4‐silapentane‐1‐sulfonic acid (DSS) as a reference. The concentration of each component was calculated from the proton NMR spectrum acquired (128 scans) with a Bruker AVIII 600 MHz NMR spectrometer (Bruker Daltonics, Billerica, MA) under standard quantitative conditions.

*Hydrogel Formation*: The RLP‐Ac and PEG‐4Ac were dissolved into PBS separately at 10 wt% (w/v) concentration. A stock solution of the photoinitiator LAP was prepared in PBS at a concentration of 13.4 mg mL^−1^. The RLP‐Ac and PEG‐4Ac stock solutions were mixed at 50/50 RLP‐Ac/PEG‐4Ac mass ratios at room temperature. 5 μL of LAP solution was added to 100 μL RLP‐Ac/PEG‐4Ac mixture and vertex mixed the samples to obtain 2.2 × 10^−3^ m LAP in the precursor mixture. An UVP Blak‐Ray B‐100AP high intensity UV lamp (UVP, Upland, CA) with 365 nm wavelength ≈5 mW cm^−2^ intensity was irradiated for 4 min on the hydrogels in 0, 5, and 10 min after vertex mixing. The UV intensity was confirmed with a radiometer.

*Oscillatory Rheology*: The oscillatory rheology experiments were conducted on an AR‐G2 rheometer (TA Instruments, New Castle, DE) with an attached UV Light Guide accessory and UV lamp source (OmniCure S2000 (Excelitas)), with an 8 mm diameter stainless steel parallel‐plate geometry. The precursor solutions were prepared as described above. The 10 μL hydrogel precursor solution was deposited on the quartz rheometer stage and the geometry was set at a 200 µm gap. Mineral oil was used to seal the geometry and prevented dehydration of the hydrogel. The 365 nm UV with 5 mW cm^−2^ intensity was applied in 0, 5, or 10 min to induce UV crosslinking. The mechanical properties of the hydrogels were measured in the linear viscoelastic regime where the modulus is independent of the level of applied stress or strain. The gelation of hydrogels was monitored using a time sweep conducted in the linear viscoelastic regime at 1% strain and an angular frequency of 6 rad s^−1^. This experiment was followed with a frequency sweep from 0.1 to 100 rad s^−1^ conducted at 1% strain and amplitude sweep from 0.1% to 1000% strain. Experiments were repeated on three samples for each condition and the shear modulus reported as the simple mean. The error is reported as the standard deviation of the samples tested.

*Polymerization Yield*: The precursor solutions were prepared in deionized water and a 20 μL hydrogel precursor solution was crosslinked with UV irradiation for either 2 or 4 min. The hydrogels was then incubated with 400 μL of deionized water for 2 h. The water was then removed from the hydrogels and lyophilized both supernate and hydrogels separately.

*Confocal Microscopy and Domain Diameter Analysis*: Hydrogel disks were formed in silicone chambers with 5 mm diameter and 0.5 mm thickness, by the same methods above, for confocal microscopy analysis of domain sizes. The confocal Z‐stack images were acquired with a Zeiss 780 multiphoton microscope (Carl Zeiss, Inc., Thornwood, NY). A Chameleon Vision II Multiphoton laser with a 755 nm wavelength was used to excite the autoflorescence of the RLP, and the NDD detection system was used for imaging the multiphoton florescence. The domain diameters were analyzed with Volocity software.

*BCARS Imaging*: The custom‐built BCARS setup has been described in detail by Billecke et al.^98^ Briefly, a commercial laser source (Leukos‐CARS, Leukos) delivered 1 ns pulses of a 1064 nm laser with 32 kHz repetition rate which was split to serve as pump/probe beam and to pump a photonic crystal fiber generating a supercontinuum (>100 µW nm^−1^, 1100–1600 nm) employed as the Stokes beam. Both beams were overlapped in the focal plane of an inverted microscope (Eclipse Ti‐U, Nikon) equipped with an *xyz* piezostage (Nano‐PDQ 375 HS, Mad City Labs) using a 100×, 0.85 NA (Olympus) objective. The spectral signal was collected in forward direction via an air objective (M‐10×, 0.25 NA, Newport), where the laser lines were removed with a notch (NF03‐532/1064E‐25, Semrock) and a shortpass filter (FES1000, Thorlabs) before being dispersed by a spectrometer (Shamrock 303i, 300 lines mm^−1^, 1000 nm blaze, Andor), and finally collected on a deep‐depletion CCD (Newton, DU920P‐BR‐DD, Andor). Several stitched images (4 tiles, 250 nm steps, 101 × 101 pixels per tile) with 500 ms pixel dwell time were collected per sample with a spectral range between 600 and 3400 cm^−1^.

*BCARS Data Analysis*: Raw spectra were treated with a modified Kramers–Kronig algorithm^99^, ^100^ and an error phase correction via an interactive noise‐maintained approach^101^ made model‐free using a second‐order Savitzky–Golay filter (404 cm^−1^ window size) to remove nonresonant contributions and to generate a Raman‐like spectrum from the imaginary component of the third‐order susceptibility. Data analysis as well as the generation of images for specific Raman frequencies was done in IgorPro 6.34 (Wavemetrics). Images of the PEG‐ and RLP‐rich phases were generated by integrating over 10 wavenumbers around 1468 and 1660 cm^−1^ and then dividing the two images. These images were then used to identify ten regions within each phase, where spectra were extracted with a 3 × 3 pixel bin. Then the region between 1350 and 1700 cm^−1^ was fitted in Matlab (Mathworks) with a sum of Lorentzian functions as summarized in **Table** **2** and a ratio of the respective peak areas were formed to follow the composition of the two phases with crosslinking time.

**Table 2:** Fitting Parameters used on Raman‐like spectra. Seed values with lower/upper bounds

Peak position [cm^−1^]	FWHM [cm^−1^]	Vibration
1384 (1382–1386)	12 (10–15)	CH_3_ 104
1412 (1408–1416)	12 (10–15)	CH_2_ wagging^105^
1440 (1435–1445)	12 (10–15)	CH_2_ deformation^104^
1468 (1460–1470)	12 (10–15)	CH_2_ bending^87^
1607 (1600–1615)	12 (10–15)	Ring modes phenylalanine^104^
1666 (1650–1670)	12 (10–40)	amide I^105^

*AFM Indentation*: Force and mechanical measurements were acquired using a Bruker Catalyst AFM. The micromechanical properties of RLP/PEG hydrogels were characterized via indentation (AFM) with a tip equipped with a 1 µm polystyrene probe, with a spring force constant of 0.09 N m^−1^ (NovaScan). The hydrogel was preformed between two glass slides (equipped with a 40 µm spacer) using the cross‐linking protocol described in the sections above. The thin hydrogels were hydrated in PBS buffer prior to the experiment; the hydrated hydrogel thickness was ≈40–60 µm. The hydrogels were indented with the AFM probe until the force reached a 1 nN threshold, and the Young's modulus was evaluated from fitting the force curve data to the Hertzian Spherical model.^102^, ^103^, ^106^ The Poisson's ratio was assumed to be 0.5 for the hydrogel due to the high water content and the incompressibility of the hydrogel samples.^91^ Over 100 indentations for each of three replicates were performed; analysis of the Young's modulus from the data was conducted with the NanoScope Analysis software. The Bruker Catalyst AFM was mounted onto a Zeiss AxioObserver inverted microscope to perform simultaneous bright‐field microscopy and force spectroscopy; the light microscope allowed accurate positioning of the AFM probe on individual microdomains and other hydrogel features.

*Swelling Ratio*: The precursor solutions were prepared as described above. Hydrogels were then incubated with PBS overnight. The weight of swollen hydrogels (*W*_s_) was recorded, and the hydrogels were then washed with deionized water three times and lyophilized to obtain the dry weight (*W*_d_). The swelling ratios were calculated as *W*_s_/*W*_d_.

*Cell Encapsulation and Viability*: The hMSCs (Lonza, MD) were encapsulated in 10 wt% 50/50 RLP‐MMP‐RGD‐2Ac/PEG‐4Ac hydrogels at a cell density of 1 × 10^6^ cells mL^−1^ (hMSC). The precursors were vortex mixed and then pipette mixed with the cells before being deposited onto silicone chambers that were 5 mm in diameter and 1 mm in thickness, which were then placed under a 365 nm UV lamp (UVP, Upland, CA) and irradiated at 5 mW cm^−2^ for 2 min. The hydrogels were then placed into the cell growth media from the MSCGM bullet kit for (hMSC, Lonza, MD) and placed in a cell culture incubator at 37 °C, 5% CO^2^. Live/Dead stain (LifeTechnologies) was utilized to determine the viability of the hMSCs. The hydrogels were washed in PBS and placed in PBS containing 2 × 10^−3^ m Calcein AM and 4 × 10^−3^ m ethidium homodimer‐1 for 20 min at 37 °C, 5% CO^2^. Cells were then washed twice with PBS and imaged while still alive. The cell–gels were then imaged via laser scanning confocal microscopy on a Zeiss LSM 710 microscope (Carl Zeiss, Inc., Thornwood, NY). The excitation (Ex) and emission (Em) waveleght of Calcein AM (Ex 488 nm/Em 490–552 nm), ethidium homodimer‐1 (Ex 561 nm/Em 560–735 nm). Z‐stack images were acquired from hydrogels and representative images in maximum intensity projections are reported.

*Statistical Analysis*: Statistical analysis was performed using the ANOVA Tukey‐Kramer HSD test with JMP Pro software, and the threshold for statistical significance was set at *p* < 0.01.

## Conflict of Interest

The authors declare no conflict of interest.

## Supporting information

Supplementary
