# Supplementary material for: Microstructured Elastomer‐PEG Hydrogels via Kinetic Capture of Aqueous Liquid–Liquid Phase Separation
Source: Adv Sci (Weinh). 2018 Mar 12;5(6):1701010. doi: 10.1002/advs.201701010 (PMC6010786; doi:10.1002/advs.201701010)
Supplement: Supplementary file 1 — Supplementary [file ADVS-5-1701010-s001.pdf]

Copyright WILEY-VCH Verlag GmbH & Co. KGaA, 69469 Weinheim, Germany, 2018.

ADVANCED SCIENCE
Open Access

Supporting Information

for *Adv. Sci.,* DOI: 10.1002/advs.201701010

Microstructured Elastomer-PEG Hydrogels via Kinetic Capture of Aqueous Liquid–Liquid Phase Separation

*Hang Kuen Lau, Alexandra Paul, Ishnoor Sidhu, Linqing Li, Chandran R. Sabanayagam, Sapun H. Parekh, and Kristi L. Kiick**

Copyright WILEY-VCH Verlag GmbH & Co. KGaA, 69469 Weinheim, Germany, 2016.

Supporting Information

Microstructured Elastomer-PEG Hydrogels via Kinetic Capture of Aqueous Liquid-Liquid Phase Separation

*Hang Kuen Lau, Alexandra Paul, Ishnoor Sidhu, Linqing Li, Chandran R. Sabanayagam, Sapun H. Parekh, and Kristi L. Kiick**

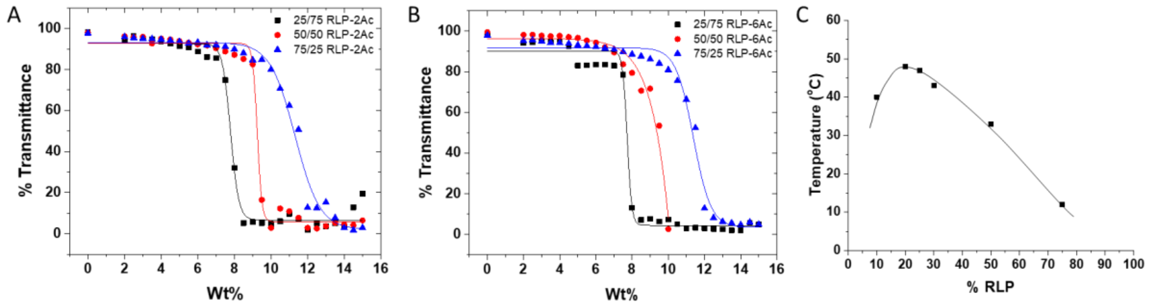

Figure S1 Phase separation of RLP-Ac/PEG-Ac in PBS buffer. UV-Vis transmittance of RLP-Ac/PEG-4Ac solutions with increasing RLP-Ac/PEG-4Ac ratios with A) RLP-2Ac and B) RLP-6Ac as a function of increasing total polymer wt%. C) Transition temperature of a 10wt% solution of RLP-6Ac/PEG-4Ac with increasing amount of RLP-6Ac.

Table S1 Turbidity measurements (UV-Vis spectroscopy) for determination of the phase separation concentrations of 10wt% 50/50 RLP-Ac/PEG-4Ac

	Cloud Point (wt%)	
RLP-2Ac	7.91	+/-0.90
RLP-4Ac	6.13	+/-0.14
RLP-6Ac	8.71	+/-0.86
RLP-10Ac	9.62	+/-0.06

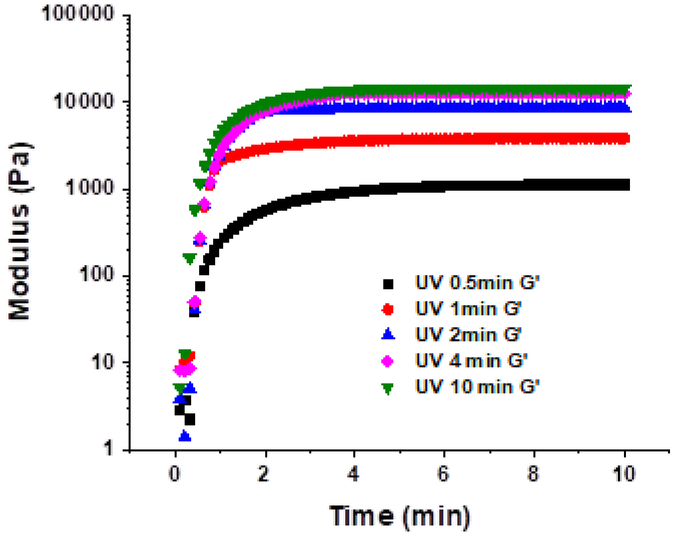

Figure S2 Modulation of hydrogel mechanical properties with variations in the time of irradiation. Data shown are oscillatory rheology time sweeps of 10 wt% 50/50 RLP-6Ac/PEG-4Ac. All samples were monitored for 10 minutes, but the various samples were irradiated with UV-light for different durations starting at time 0 (e.g., irradiation for 30 sec, 1 min, 2 min, 4 min and 10 min), illustrating the control over mechanical properties that is afforded by these methods.

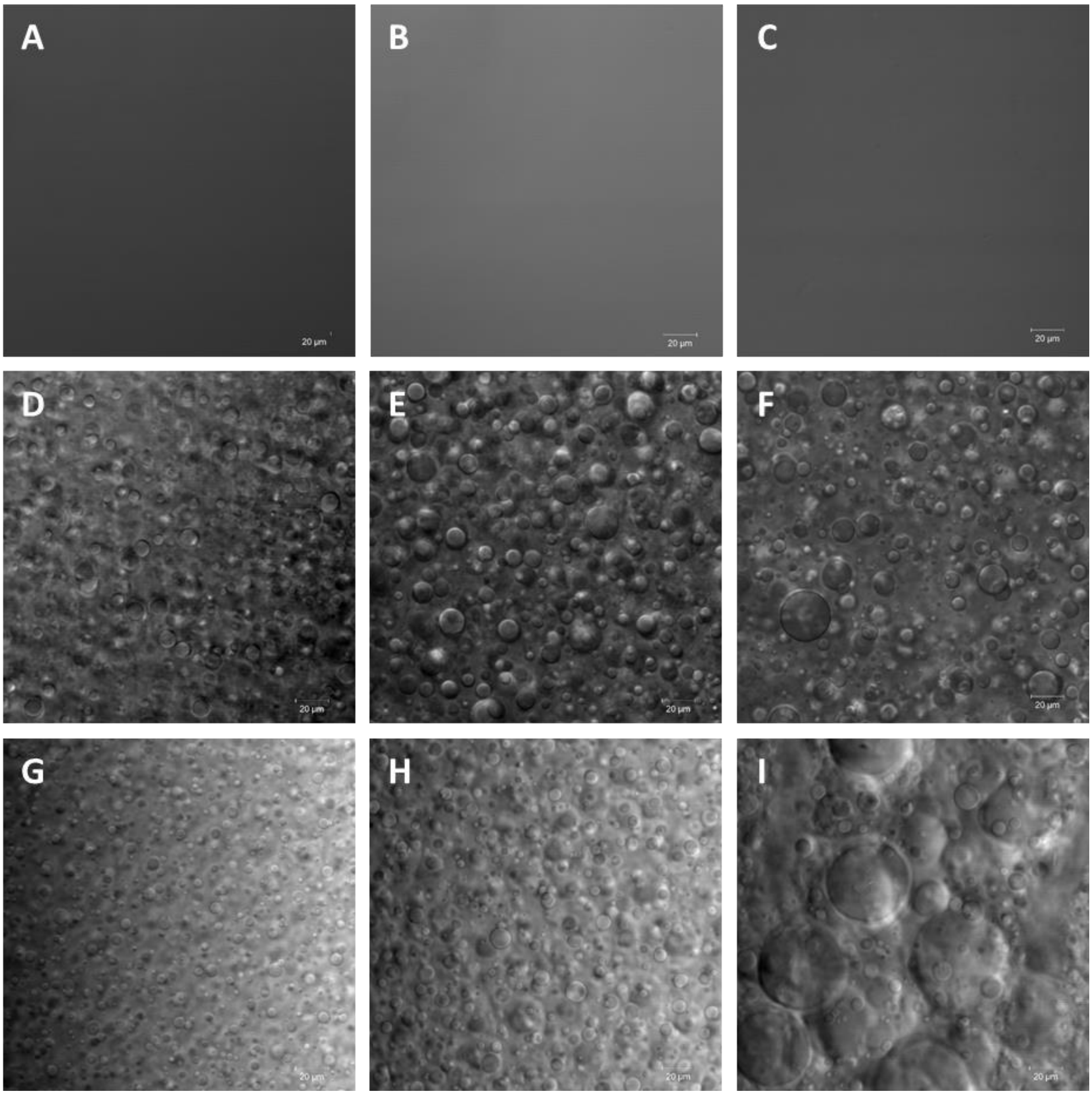

Figure S3 Phase contrast images of photo-crosslinked RLP and PEG hydrogels. A) 10 wt% PEG-4Ac, B) 10 wt% RLP-2Ac and C) 10 wt% RLP-6Ac hydrogels crosslinked immediately after mixing with UV irradiation for 4 min. The lack of contrast observed in these experiments indicates the absence of microstructure in pure PEG and RLP hydrogels (A-C). (D- F) 10 wt% 50/50 RLP-6Ac/PEG-4Ac and (G-I)10 wt% 50/50 RLP-2Ac/PEG-4Ac hydrogels UV irradiation at (D, G) 0, (E, H) 5 and (F, I) 10 min after mixing. Samples in panels D through I were also crosslinked with UV irradiation for 4 min.

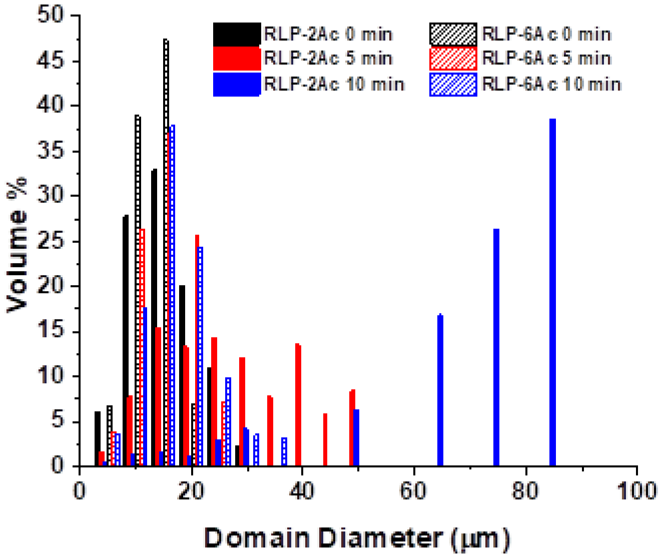

Figure S4 Domain diameter distribution of the RLP-rich domains, with different times of incubation prior to photocrosslinking of RLP-PEG hydrogels. Data are shown for 50/50 10 wt% hydrogels comprising either RLP-2Ac or RLP-6Ac.

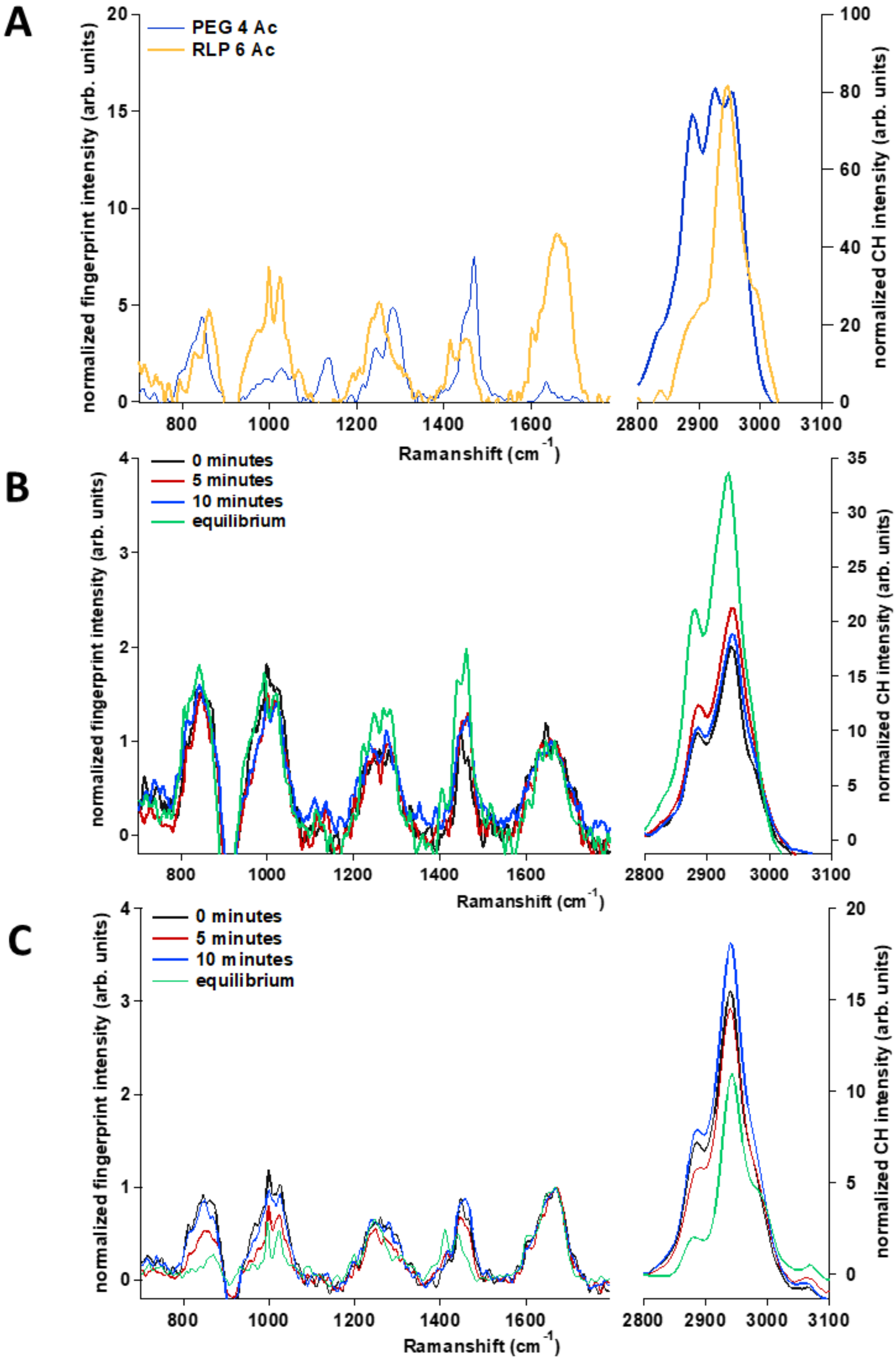

Figure S5 CARS spectra A) of 10wt% RLP-6Ac and PEG-4Ac in PBS, B) the PEG-rich phase and C) the RLP-rich phase in the hydrogels. Spectra in B and C are normalized by the maximum value in the amide I region.

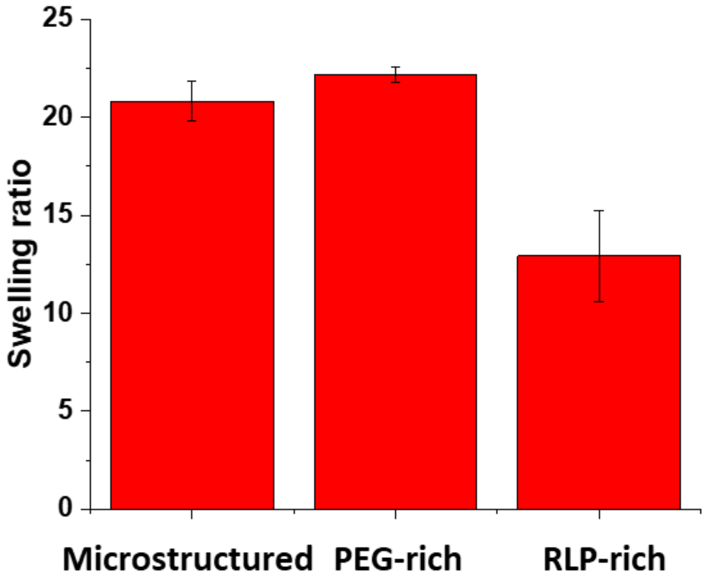

Figure S6 Swelling ratios for the microstructured hydrogels and also for hydrogels formed from the individual PEG-rich and RLP-rich phases after bulk phase separation. The bulk phase-separated solutions were isolated and crosslinked into hydrogels in separate samples prior to the measurement of swelling ratios.

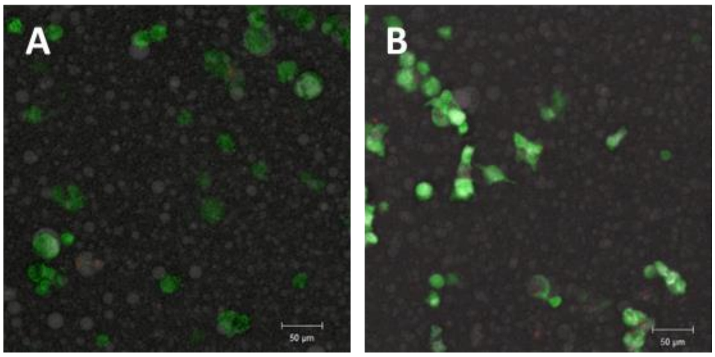

Figure S7 Cytocompatability of 10wt% RLP-MMP-RGD-6Ac/PEG-4Ac hydrogels. Confocal z-stack maximum intensity projections images for 3D cultures of encapsulated hMSCs in 10wt% RLP-MMP-RGD-6Ac/PEG-4Ac hydrogels at A) day 1 and B) day 7. Colors indicate live cells (calcein, green), dead cells (ethidium homodimer, red), and autofluorescence of RLP-rich domains (grey/white).
